# Natural history of spontaneous aortic intramural hematoma progression: Six years follow-up with cardiovascular magnetic resonance

**DOI:** 10.1186/1532-429X-12-27

**Published:** 2010-05-13

**Authors:** Xiaohai Ma, Zhaoqi Zhang, Zhanming Fan, Lei Zhao, Jing Yu

**Affiliations:** 1 Deptartment of Radiology, Beijing Anzhen Hospital, Capital Medical University, Beijing, 100029, China

## Abstract

We described a 6 years follow-up of a spontaneous aortic intramural hematoma (IMH) with cardiovascular magnetic resonance (CMR) examination. Since multiple factors may play roles in the natural history of IMH, the patient experienced the course of progression, which included hematoma absorption, ulcer-like lesion, aneurysm and limited dissection. The initial and follow-up CMR examination included 3D CE MRA and non-enhanced "bright blood" pulse sequence. The inherent advantage of outstanding contrast with plain scan, which shorten the scan time and avoid potential risk of contrast agent, might make the fast gradient echo sequence as an alternative method when following stable IMH.

## Background

Spontaneous aortic intramural hematoma (IMH), which first described in 1920 by Krukenberg as "dissection without intimal tear", results from the spontaneous rupture of the vasa vasorum of the aortic wall. IMH most frequently involves the ascending aorta (type A) or proximal descending aorta (type B). This condition presents clinically as severe chest pain radiating to the back, which is similar to aortic dissection (AD). Systemic hypertension is the leading risk factor for spontaneous IMH. Recent advances in imaging techniques have significantly improved the diagnostic accuracy and enhanced clinical understanding of IMH. The natural history of spontaneous IMH is different from classical AD and its time course of IMH can vary significantly, so early diagnosis and close follow-up is desirable. We describe a spontaneous IMH case followed for 6 years by cardiovascular magnetic resonance (CMR). The patient progressed through different stages, including hematoma absorption, ulcer-like lesion emergence, aneurysm enlargement and limited AD.

## Case History

A 65-year-old female with a long history of hypertension was referred to emergency room because of acute chest pain that persisted over one day. The echocardiogram showed a widening of the lumen of the ascending aorta. A thoracic aorta CMR examination (Siemens Sonata 1.5T, Erlangen, Germany) was performed for further evaluation. The imaging protocol included three-dimension contrast-enhanced magnetic resonance angiography (3D CE MRA, TR/TE 2.2/0.8; FOV 320 × 380 mm; effective thickness 1.23 ± 1.60 mm) and true fast imaging steady-state precession (true FISP) MRI (TR/TE 3.2/1.6 ms; field of view (FOV), 300 × 400 mm; matrix, 300 × 512; thickness, 6 mm). The true FISP images were acquired without cardiac gating. A stack of two-dimensional images were acquired in approximately 15~18 seconds. Each acquisition had 10 slices. Gadopentetate dimeglumine (0.1 mmol/kg, Magnevist, Bayer-Schering, Germany) was administrated intravenously followed by 20 ml saline flush.

**Figure 1:**
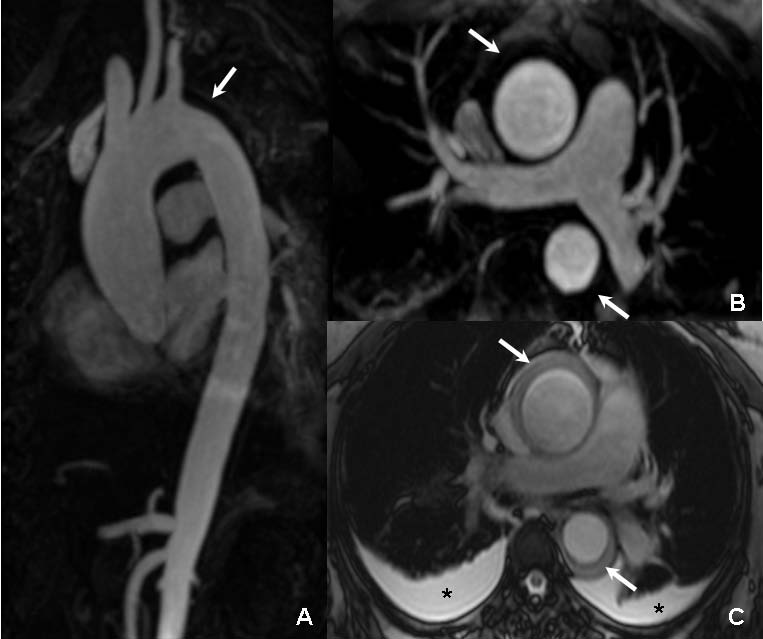
**Initial CMR: the MIP (A) and MPR (B) images of 3D CE MRA revealed dilation of the ascending aorta and a low signal intensity non-enhanced lesion (arrow) around the ascending and descending aorta**. In the true FISP axial images (C), the comparatively lower signal could be found eccentrically around both ascending and descending aorta (arrow), which was considered as hematoma. In addition, bilateral pleural effusion (asterisk) was documented.

**Figure 2:**
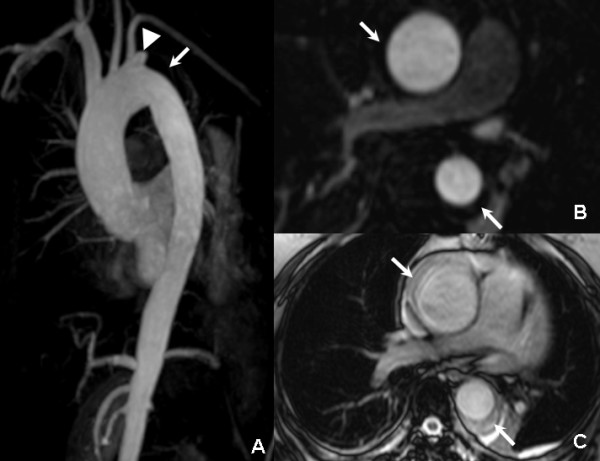
**One month follow-up CMR: the MIP (A) and MPR (B) images revealed that the non-enhanced hematoma (arrow) became more stable compared to baseline, while an ulcer-projection lesion (UPL) was found nearby the left subclavian artery orifice**. The true FISP axial images (C) were consistent with these findings and also indicated that the pleural effusion was partially absorbed.

**Figure 3:**
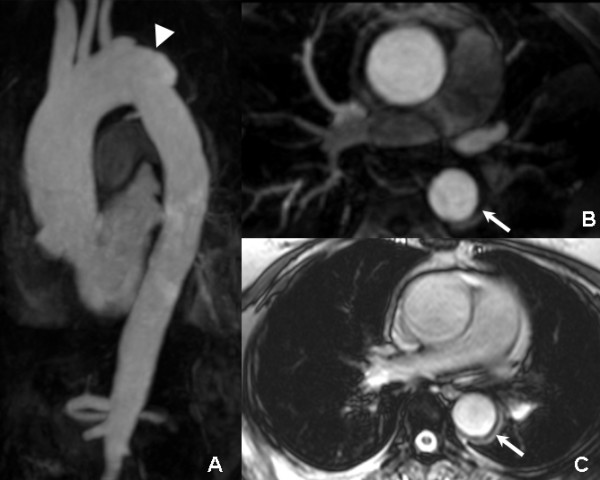
**Three months follow-up CMR: the MIP (A), MPR (B), and true FISP axial images (C) revealed partial regression of the hematoma (arrow) and complete absorption of the pleural effusion, while the ulcer-projection lesion progressed into an aneurysm like contour (arrowhead)**.

**Figure 4:**
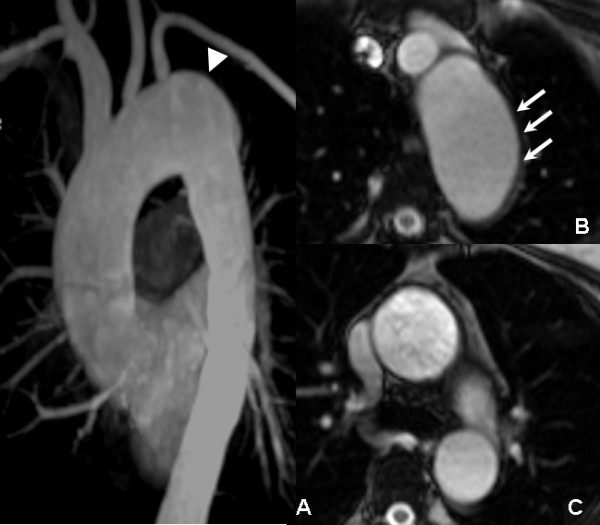
**One year follow-up CMR: the MIP (A), MPR (B), and true FISP axial images (C) revealed that the aortic arch aneurysm (arrowhead) was larger than it was at the 3 months follow-up examination**. A thickened aortic wall without an apparent low signal non-enhanced hematoma could be seen, which suggested the IMH was reabsorbed.

**Figure 5:**
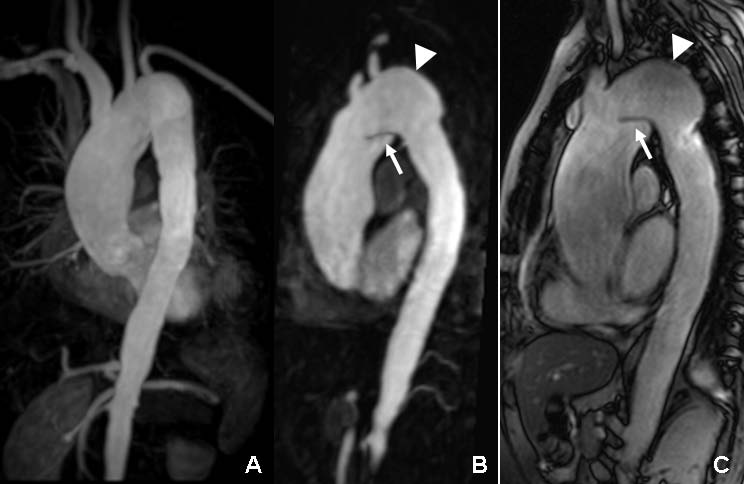
**Six year follow-up CMR: the MIP (A) image showed the extended widening lumen of ascending aorta and aortic arch with irregular wall**. The MPR (B) and true FISP sagittal (C) images displayed the luminal intimal flap (arrow) located at aortic arch that formed limited aortic dissection. The aortic arch aneurysm was still present (arrowhead).

An eccentric non-enhanced low signal intensity lesion around the widened ascending (50 mm) and descending aorta were seen on the reconstructed 3D CE MRA and true FISP axial images. Aortic IMH (type A) combined with bilateral pleural effusion was documented (Figure 1). The symptoms ceased after conservative medical treatment. Short-term follow-up CMR examination was repeated at 1 month (Figure 2) and 3 months (Figure 3) after initial onset. The hematoma was stable at 1 month and partially absorbed at 3 months. The pleural effusion resolved entirely during the 3 months period. However, an ulcer-projection lesion (UPL) was found immediately distal to the origin of the left subclavian artery at 1 month. At 3 months, the UPL had become larger and progressed into an aneurysm-like lesion. Since the patient was asymptomatic during this period, she refused surgery or intervention. At I year follow-up, CMR showed that the diameter of the ascending aorta had increased (from 50 mm to 57 mm) and that the aortic arch aneurysm had grown (Figure 4). Six years later, a luminal intimal flap was found at the ascending-arch of the aorta and diagnosed as limited AD (Figure 5).

## Discussion

There are controversial issues exist in indication of treatment of spontaneous IMH. Most studies currently recommend early surgery or interventional therapy for patients with proximal IMH and medical management for patients with distal IMH; this is similar to that of classical AD. However, others favor a conservative treatment, with initial medical management for all patients in a stable condition. Multiple factors may play roles in the natural history of spontaneous IMH, including blood pressure, vessel condition, hemodynamic changes, the wall thickness and diameter of the aorta, and even age and race [1,2]. Moizumi et al [3] demonstrated that the aorta-related events occur equally in both types of IMH. Kaji et al [4] showed that maximum aortic diameter is predictive of progression for type A IMH with an optimal cutoff value of about 50 mm. Nishigami et al [5] reported that 18% of IMHs disappeared within 1 month and 48% within 6 months. Furthermore, those patients with an IMH that resolved or a maximal aortic diameter of <45 mm suggest good prognosis. A potential reason for progression is that aortic IMH can weaken the medial layer of aorta and increase the likelihood of fusiform aortic aneurysm or AD, especially in those with high aortic pressure or a newly emerged ulcer-like lesion.

Based on short-term (<30 days) follow-up, several studies have suggested that the natural history of IMH is a dynamic process which can result in reabsorption, classical AD, or aortic rupture. Granha et al [6] reported that IMH with ulcer-like a lesion was significantly associated with disease progression, whereas IMH without an ulcer-like lesion had a reliable stable course. In a long-term study (mean follow-up 45 months) with 68 cases, Evangelista et al [7] found that IMH most frequently evolved into aortic aneurysm or pseudoaneurysm, while complete regression without changes in the size of the aorta was observed in 30% cases. Progression to classical dissection was less common. In our 6 years of follow-up, the patient's blood pressure remained stable and no suspected symptoms occurred. However, the course of spontaneous IMH experienced hematoma absorption, ulcer-like lesion emergence, and aneurysm enlargement in the short-term and limited AD in the long-term. It verified that multiple factors may play roles in the course of disease.

As a noninvasive imaging modality that does not require ionizing radiation, CMR has been preferred for the purposes of diagnosis and follow-up for IMH during the past decade [8]. The 3D CE MRA has been considered as one of the most effective techniques to diagnose aortic diseases. When combined with suitable post-processing, it can be used to differentiate primary IMH from classical AD and IMH associated with penetrating atherosclerotic ulcer (PAU). CE-MRA is a good tool for looking at the lumen but less effective at positively identifying mural haematoma, which may be missed by the unwary. Besides, emergency CMR evaluation for aortic injury or disease has not been considered practical due to prolonged examination times, especially after the rapid development of multi-detector computed tomography (CT), while CT is not suitable for long term follow-up medically stable patients because of radiation risk and the potential side effects of the associated contrast agents.

Thus, researchers have focused on non-enhanced fast CMR imaging sequences to address the scan time limitations. Pereles et al [9] evaluated the fast gradient echo sequence termed as trueFISP, which demonstrates good contrast of the fluid flow, and shortens the examination time to less than 4 minutes. The accuracy of diagnosis for the presence of aortic dissection and aortic aneurysm was 100%. In our case, the true FISP sequence was applied in the patient's initial and follow-up examinations. The trueFISP axial and/or sagittal images showed the crescent or ring- like thickening of the aortic wall due to the IMH, and displayed the low signal ring which corresponds to the intima between the aortic lumen and hematoma. The true FISP is also useful for detecting some of the complications of aortic diseases, such as pleural effusion and pericardial effusion, which is helpful in predicting the prognosis. The use of true FISP imaging is done using a non-ECG gated sequence with multiple slices, which is not ideal for evaluation of wall thickness or positively identifying haematoma (vs. laminated thrombus in an aneurysm). Tissue characterisation with ECG gated black-blood images may have been more helpful.

In conclusion, the inherent contrast features of the fast gradient echo sequence without the use of a contrast agent make it a potential alternative when following stable IMH.

## Competing interests

The authors declare that they have no competing interests.

## Authors' contributions

XA: Literature research, manuscript preparation and editing. ZZ: Case collection. ZF: Case collection. LZ: Manuscript preparation and editing. JY: Picture and word editing.

All authors read and approved the final manuscript.
